# Comparison of life quality of pregnant adolescents with that of pregnant adults in Turkey

**DOI:** 10.3109/03009731003628724

**Published:** 2010-10-27

**Authors:** Semiha Taşdemir, Balci, Osman Günay

**Affiliations:** ^1^Kizilay Hospital, KayseriTurkey; ^2^Erciyes University Medical Faculty, Department of Public Health, KayseriTurkey

**Keywords:** Adolescent, adult, life quality, pregnancy

## Abstract

**Objectives:**

This study aimed to determine the quality of life of pregnant adolescents aged < 20 years and pregnant adults aged between 20–29 years, to evaluate the effects of gestational periods on the quality of life, and to compare the quality of life scores of pregnant adolescents and adults.

**Methods:**

This study was performed in Turkey in 2007. Totally, 147 pregnant adolescents aged < 20 years and 156 pregnant adults aged between 20 and 29 years were included. A questionnaire on socio-demographic and obstetric characteristics was administered by face-to-face interviewing method, and Short Form-36 scale was applied.

**Results:**

The mean quality of life scores ranged between 44.2 and 56.1 points for the adolescents and between 44.6 and 59.9 points for the adults. All quality of life scores, except bodily pain, were lower for adolescents than for adults. It was determined that the quality of life scores in pregnancy were generally lower in the first trimester, significantly increased in the second trimester, and decreased to the lowest level in the third trimester.

**Conclusions:**

Quality of life scores of the pregnant adolescents were significantly lower than the pregnant adults. Physical care, support, and education programs may be beneficial to increase the quality of life levels in pregnancy.

## Introduction

The quality of life of women may be negatively affected by a number of changes experienced during the pregnancy period. Pregnancy-related physical symptoms include fatigue, nausea, vomiting, heartburn, leg cramps, hemorrhoids, and shortness of breath (1), many of which have potentially negative effects on women's lives during pregnancy. For this reason, the quality of life of pregnant women is expected to be lower than that of non-pregnant women of the same age.

Pregnant adolescents encounter challenges in addition to those encountered by all women who are pregnant. Pregnant adolescents are at greater risk for certain health conditions, such as pregnancy-induced hypertension, pre-eclampsia, intra-uterine growth retardation, preterm delivery, low birth-weight, and inadequate weight gain, related to their age and developmental stage, and have limited knowledge of their bodies, reproduction, pregnancy, and birth (2–7). Additionally, pregnant adolescents have been found to be at higher risk when compared to pregnant adults for certain social problems, such as discontinuation of education, unemployment, and social isolation (2,6). In cases in which a woman has not completed her physical, psychological, and social development, the pregnancy period affects the mother and infant negatively. Thus, the quality of life of pregnant adolescents is expected to be lower than that of pregnant adults. In many parts of the world, programs are directed in order to prevent adolescent pregnancy, which is an important risk factor for mortality and morbidity (8).

The Medical Outcomes Study 36-Item Short-Form Health Survey (SF-36) is a generic scale developed by Ware and Sherbourne (9). The SF-36 is a general, short, but comprehensive health questionnaire appropriate for clinical practice and population surveys, and the SF-36 has strong psychometric aspects. There are eight subscales of the SF-36, which are physical functioning, role–physical, bodily pain, general health, vitality, social functioning, role–emotional, and mental health. These subscales measure different aspects of health-related quality of life. The scores for each subscale vary between 0 and 100. Higher scores show better quality of life. There is no cut-off point.

The SF-36 can be used for every age, disease, and treatment group to compare the effects of the disease and the benefits of different treatments (10). A number of studies point out that the SF-36 scale may be used in different diseases to measure the quality of life (11,12). The scale is also used to evaluate the quality of life of pregnant women (13). The Turkish version of SF-36 has been validated by Koçyigit et al. (14).

There are a limited number of studies on the quality of life of pregnant women and the effects of several factors on the quality of life (15). The number of studies related to the effects of adolescent pregnancy on quality of life is also limited (16).

The aim of the study was to determine the quality of life of pregnant adolescents < 20 years of age and pregnant adults between 20 and 29 years of age, to evaluate the effects of gestational periods on the quality of life, and to compare the quality of life scores of pregnant adolescents and pregnant adults.

## Material and methods

A cross-sectional study design was used, and data were collected in 2007 in the provincial center of Kayseri, Turkey.

In the current study, two groups consisting of equal sample sizes of pregnant adolescents and adults were planned. The quality of life score difference between the pregnant adolescents and adults was assumed to be 5 points, and the standard deviation of the quality of life scores was assumed to be 15. With a confidence level of 0.95 and a power of 0.80, the minimum sample size was calculated to be 142 for both groups. Therefore, 180 women were included in each group.

A multistage sampling method was used. In the first stage, 6 of 40 primary health centers in the provincial center of Kayseri were selected randomly. The total population of the selected health centers was approximately 185,000. In the second stage, obstetric follow-up records in the primary health centers were examined. It was determined that there were 219 pregnant women who were younger than 20 years of age and 1258 pregnant women between 20 and 29 years of age. Pregnant adolescents (< 20 years of age) and pregnant adults (between 20 and 29 years of age) were listed. A total of 180 pregnant adolescents and 180 pregnant adults were selected through simple random sampling method.

All the pregnant women in the sample were visited at their home by the authors. For the women who were absent at the visit date, their neighbors and relatives were informed, and they were revisited a week later. A total of 33 adolescents and 24 adults who could not be reached in spite of two visits were not included in the study. All of the pregnant women who were reached agreed to participate in the present study. Therefore, 147 pregnant adolescents (81.7%) and 156 pregnant adults (86.7%) were included after verbal informed consent was obtained.

### Data collection

The study data were collected in 2007. A questionnaire consisting of 36 questions on socio-demographics and fertility characteristics was completed by face-to-face interviewing method. After completion of the questionnaire, the SF-36 scale was administered. The pregnant women completed the SF-36 scale under the supervision of the authors. The investigators aided the women having difficulties completing the scale.

The SF-36 scores were determined along with the calculation of eight subscale scores of quality of life in accordance with the instructions. The scores obtained from the subscales of the quality of life could vary between 0 and 100, with higher scores representing a better quality of life.

### Ethical considerations

The study was approved by the Ethical Committee of Erciyes University Medical Faculty. After the participants were informed about the study design, individual verbal informed consent was obtained.

### Data analysis

The obtained data were analyzed with the SPSS 13.0 statistical software package (SPSS Inc., Chicago, IL, USA). Quantitative data were expressed as mean ± standard error (mean ± SEM). For statistical comparisons, Pearson's chi-square test was performed for categorical variables, and unpaired *t* test and general linear model (GLM) were performed for quantitative data. In determination of the impact of age groups and gestational periods on quality of life scores, economic status, educational status, and the total number of pregnancies were taken as covariates. Statistical significance was accepted as *P* < 0.05.

## Results

The present study consisted of two groups. The first group was comprised of 147 pregnant adolescents < 20 years of age, and the second group was comprised of 156 pregnant adults between 20 and 29 years of age. All of the women in both groups were married. The mean age at first marriage was 16.9 ± 1.3 and 20.2 ± 3.5 years, in the pregnant adolescents and adults, respectively. The socio-demographic and obstetric characteristics of these groups are demonstrated in Table I. The mean age, the age at first marriage, and the multiparity of the pregnant adolescents were lower than of the pregnant adults. The proportion of employed women and those with a good economic status among the pregnant adolescents were lower than in the adult group (*P* < 0.001). No significant differences were detected between the other socio-demographic and obstetric characteristics of the adolescent and adult groups.

**Table I.** Socio-demographics and obstetric characteristics of pregnant adolescents and adults.

	Age groups		
Characteristics	Adolescent (*n* = 147)	Adult (*n* = 156)	*P*^a^
Age, year (mean ± SEM)	18.0 ± 0.1	23.9 ± 0.3	
Age at first marriage, year (mean ± SEM)	16.9 ± 0.1	20.2 ± 0.3	< 0.001
Multiparity, *n* (%)	67 (42.9)	89 (57.1)	0.029
Unintended pregnancy, *n* (%)	85 (57.8)	65 (41.7)	0.050
Gestational period			
1st trimester, *n* (%)	47 (32.0)	53 (34.0)	0.932
2nd trimester, *n* (%)	48 (32.7)	49 (31.4)	
3rd trimester, *n* (%)	52 (35.4)	54 (34.6)	
Consanguineous marriages, *n* (%)	29 (19.7)	21 (13.5)	0.142
Social insurance, *n* (%)	136 (92.5)	147 (94.2)	0.548
Smoking, *n* (%)	12 (8.2)	17 (10.9)	0.419
Education (secondary school and above), *n* (%)	65 (44.2)	84 (53.8)	0.094
Occupation (employed), *n* (%)	6 (4.1)	28 (17.9)	< 0.001
Economic status			
Good, *n* (%)	21 (14.3)	52 (33.3)	< 0.001
Moderate, *n* (%)	73 (49.7)	66 (42.3)	
Poor, *n* (%)	53 (36.1)	38 (24.4)	

^a^The *P-*values were calculated by using unpaired *t* test and chi-square test.

The quality of life scores of both the adolescent and adult groups are given in Table II. As seen in the table, all quality of life scores, except the bodily pain scale, were lower among the adolescents when compared to the adults.

**Table II.** Comparison of quality of life scores of pregnant adolescents and adults.

	Age Groups		
SF-36 Scale	Adolescent (*n* = 147) (mean ± SEM)^a^	Adult (*n* = 156) (mean ± SEM)^a^	*P*^b^
Physical functioning	56.1 ± 1.3	59.9 ± 1.3	0.004
Role–physical	44.2 ± 3.1	54.1 ± 3.0	0.008
Bodily pain	44.3 ± 0.5	44.6 ± 0.5	0.358
General health	46.8 ± 1.4	49.2 ± 1.4	0.048
Vitality	49.2 ± 1.3	53.1 ± 1.3	0.012
Social functioning	47.2 ± 1.5	50.7 ± 1.5	0.049
Role–emotional	47.4 ± 3.2	56.3 ± 3.1	0.021
Mental health	55.0 ± 1.4	57.4 ± 1.4	0.046

^a^Values were adjusted according to the economic status, educational status, and total number of pregnancies.

^b^The *P*-values were calculated by using general linear model.

Table III shows the quality of life score comparison of the entire group according to gestational periods. All of the scales of the SF-36, except bodily pain, had significant differences between the trimesters. Except bodily pain scale, all quality of life scores were highest in the second trimester and lowest in the third trimester.

**Table III.** Comparison of quality of life scores according to the gestational periods for the entire study sample.

	Gestational periods			
SF-36 Scale	1st trimester (*n* = 100) (mean ± SEM)^d^	2nd trimester (*n* = 97)(mean ± SEM)^d^	3rd trimester (*n* = 106)(mean ± SEM)^d^	*P*^e^
Physical functioning	57.6 ± 1.6^a,b^	61.4 (1.6)^a^	54.9 (1.5)^b^	0.022
Role–physical	46.7 ± 3.7^a^	62.2 (3.7)^b^	38.7 (3.6)^c^	< 0.001
Bodily pain	45.4 ± 0.6	44.3 (0.6)	43.8 (0.6)	0.171
General health	45.8 ± 1.7^a^	54.5 (1.7)^b^	43.8 (1.6)^a^	< 0.001
Vitality	51.0 ± 1.6^a^	56.7 (1.6)^b^	45.8 (1.5)^c^	< 0.001
Social functioning	47.8 ± 1.8^a^	57.5 (1.9)^b^	41.5 (1.8)^c^	< 0.001
Role–emotional	46.8 ± 3.7^a^	63.3 (3.9)^b^	45.4 (3.7)^a^	0.002
Mental health	56.9 ± 1.7^a^	61.9 (1.7)^b^	50.0 (1.7)^c^	< 0.001

^a,b,c^The groups not having the same letter in each row were significantly different from each other.

^d^Values were adjusted according to the economic status, educational status, and total number of pregnancies (mean ± standard error).

^e^The *P*-values were calculated by using general linear model.

Table IV refers to the quality of life scores relevant to age groups and gestational periods. For both pregnant adolescents and pregnant adults, all quality of life scores, except the physical functioning and bodily pain scales, were significantly different within the gestational periods. The quality of life scores were highest in the second trimester for both groups. When the gestational periods were evaluated separately, the quality of life scores of the adolescent group were lower than in the adult group for all trimesters; however, these differences were not significant, with the exception of the role–physical (*P* = 0.037) and role–emotional (*P* = 0.048) scales for the second trimester.

**Table IV.** Comparison of quality of life scores according to the age groups and gestational periods.

		Gestational periods			
SF-36 Scale	Age groups	1st trimester	2nd trimester	3rd trimester	*P*^e^
(mean ± SEM)^d^	(mean ± SEM)^d^	(mean ± SEM)^d^
Number	Adolescent (*n*)	47	48	52	
	Adult (*n*)	53	49	54	
Physical functioning	Adolescent	54.8 (2.3)^a,b^	60.8 (2.2)^a^	50.1 (2.3)^b^	0.012
	Adult	60.4 (2.1)	62.0 (2.3)	59.9 (2.2)	0.124
	*P*	0.146	0.217	0.114		
Role–physical	Adolescent	39.4 (5.4)^a^	55.0 (5.2)^b^	38.2 (5.0)^a^	0.024
	Adult	54.0 (5.0)^a^	69.3 (5.3)^b^	39.1 (5.0)^c^	0.001
	*P*	0.047	0.037	0.894		
Bodily pain	Adolescent	44.5 (0.9)	44.0 (0.9)	44.5 (0.9)	0.968
	Adult	46.2 (0.9)	44.5 (0.9)	44.6 (0.5)	0.059
	*P*	0.138	0.788	0.176		
General health	Adolescent	42.1 (2.4)^a^	54.7 (2.4)^b^	43.7 (2.4)^a^	< 0.001
	Adult	49.4 (2.2)^a,b^	54.3 (2.4)^a^	43.8 (2.3)^b^	0.007
	*P*	0.057	0.947	0.812		
Vitality	Adolescent	48.5 (2.3)^a,b^	54.4 (2.3)^a^	44.9 (2.2)^b^	0.005
	Adult	53.5 (2.2)^a,b^	59.1 (2.3)^a^	46.8 (2.2)^b^	< 0.001
	*P*	0.143	0.133	0.684		
Social functioning	Adolescent	44.9 (2.7)^a^	55.4 (2.6)^b^	41.1 (2.6)^a^	< 0.001
	Adult	50.7 (2.5)^a^	59.7 (2.7)^b^	41.8 (2.5)^c^	< 0.001
	*P*	0.132	0.191	0.996		
Role–emotional	Adolescent	41.8 (5.6)^a^	57.5 (5.5)^b^	42.8 (5.4)^a^	0.043
	Adult	51.8 (5.2)^a^	69.1 (5.6)^b^	48.0 (5.2)^a^	0.010
	*P*	0.217	0.048	0.576		
Mental health	Adolescent	55.3 (2.5)^a,b^	60.7 (2.5)^a^	48.9 (2.4)^b^	< 0.001
	Adult	58.0 (2.4)^a,b^	63.2 (2.5)^a^	51.0 (2.3)^b^	< 0.001
	*P*	0.470	0.542	0.589	

^a,b,c^The groups not having the same letter in each row were significantly different from each other.

^d^Values were adjusted according to the economic status, educational status, and number of total pregnancies.

^e^The *P*-values were calculated by using the general linear model.

It was determined that all quality of life scores for intended pregnancies were significantly higher than unintended pregnancies in the adolescent and adult groups.

## Discussion

The Turkish Demographic and Health Survey 2003 (TDHS-2003) reported that the median age at first marriage among women between 25 and 49 years of age is 20 years. Within this group of women, the age at first marriage decreases with the increase in age, because the mean age at first marriage of women in Turkey has increased in recent years (17). However, in our study, the age at first marriage of the pregnant adolescents was lower than that of the pregnant adults.

In Turkey, the rate of extra-marital pregnancy is very low; hence all of the women included in our study sample were married. In our study, approximately 20% of adolescents and 13.5% of adults had consanguineous marriages. About one-half of the women in both groups reported their last pregnancy as unintended. The percentage of unintended pregnancies in the adolescent group was significantly higher than in the adult group. It has been known that adolescents tend to experience higher rates of unintended pregnancy than do adults (2).

In the current study, only 4.1% of the adolescents and 17.9% of the adults were employed. Self-reported economic status was good in 13.3% of the adolescents and in 33.3% of the adults. Significant differences were determined between the adolescents and adults from the standpoints of employment and economic status. These data illustrate the economic problems among the study groups and the severity of this problem, especially among adolescents. Differences in the fertility characteristics and economic status may be due to the early marriages in families with a low socio-economic level and the early pregnancies resulting from early marriages. Additionally, early marriages and early pregnancies prevent educational and employment opportunities. Moreover, difficult social situations negatively affect how some groups of women envision their role as a mother as they are unprepared for pregnancy (2).

In our study, the quality of life scores were generally low among the study groups. The mean quality of life scores ranged between 44.2 and 56.1 for adolescents and between 44.6 and 59.9 for adults. Altiparmak (18) measured the quality of life scores of pregnant women with a different scale without age discrimination and showed high scores for the pregnant women who were living in urban areas, in couples who had a good level of education, and in couples who intended to conceive. In our study, all quality of life scores for the adolescent group were lower than for the adult group, except the bodily pain scale. Drescher et al. (16) also found low quality of life scores for pregnant adolescents. These results may be due to the physical, mental, and social difficulties experienced by adolescents who become pregnant before the completion of their physical and mental maturation. Pregnant adolescents often require additional support during pregnancy and the post-partum period (19). Pregnant adolescents often need information about nutrition, normal fetal growth and development, body changes associated with pregnancy, common ailments and their treatments, and general safety during pregnancy (20). Non-judgmental care and simple instructions are essential for the care of pregnant adolescents (19). The support and health education during pregnancy may increase the quality of life levels of pregnant adolescents.

The quality of life scores differed according to the gestational periods for the study groups. When the pregnant adolescents and adults were investigated separately, the quality of life scores between the gestational periods were significantly different, with the exception of the physical functioning and bodily pain scales. In both groups, the quality of life scores, which were relatively low for the first trimester, increased significantly in the second trimester, and decreased to the lowest level in the third trimester. However, when the study group was stratified according to gestational periods, no significant differences existed between the adolescents and adults. The reason for these results is the decrease in the sample size per the gestational groups when the study group was stratified by gestational periods.

The variations in quality of life scores as a function of the gestational periods corresponded closely to the normal physiological changes due to pregnancy. For instance, complaints such as nausea, vomiting, and weakness, which were thought to affect the quality of life, were most common in the first trimester, yet decreased and disappeared spontaneously in the second trimester. Abdominal distension, constipation, urinary tract stasis and infections, muscular and skeletal system pain due to the increase in weight, anticipatory excitement toward the end of pregnancy, anxiety, and curiosity are more obvious and affect the quality of life.

The economic status, educational status, and total number of pregnancies are thought to affect the quality of life scores of women. In order to eliminate the confounding effects of economic status, educational status, and total number of pregnancies, covariance analysis was performed for the effect of age groups and gestational periods on quality of life scores. In other words, the effect of age groups and gestational periods on quality of life scores was independent of the economic status, educational status, and total number of pregnancies of the women.

This cross-sectional study had some limitations. First, the present study was carried out in an urban area and does not reflect the quality of life of the pregnant women living in rural areas. Future studies, including the rural areas where adolescent pregnancies are expected to be more common, may be beneficial. Second, a 5-point difference in quality of life scores between the two groups was assumed as significant during calculation of the sample size. As the study group was evaluated according to gestational periods, the data in each group decreased. Therefore, even a 10-point difference between the groups was not detected as significant. To assess the effect of various factors on the quality of life scores of pregnant women, a greater sample size should be used in future studies.

It was concluded that the quality of life scores of the pregnant adolescents were significantly lower than those of the pregnant adults, except the bodily pain scale. Therefore, in countries like Turkey, where nearly all the pregnancies are matrimonial, adolescent pregnancies may also be prevented by avoiding adolescent marriages. In order to increase the quality of life scores of pregnant women, especially pregnant adolescents, physical care, support, and education programs should be provided. Also, performing further longitudinal studies to determine the differences in the quality of life scores of pregnant adolescents and adults may be beneficial.
